# β-Adrenergic Stimulation Increases Cav3.1 Activity in Cardiac Myocytes through Protein Kinase A

**DOI:** 10.1371/journal.pone.0039965

**Published:** 2012-07-13

**Authors:** Yingxin Li, Fang Wang, Xiaoying Zhang, Zhao Qi, Mingxin Tang, Christopher Szeto, Ying Li, Hongyu Zhang, Xiongwen Chen

**Affiliations:** 1 Cardiovascular Research Center and Department of Physiology, Temple University School of Medicine, Philadelphia, Pennsylvania, United States of America; 2 Institute of Burn Research, Southwest Hospital, State Key Laboratory of Trauma Burns and Combined Injury, Third Military Medical University, Chongqing, China; Georgia State University, United States of America

## Abstract

The T-type Ca^2+^ channel (TTCC) plays important roles in cellular excitability and Ca^2+^ regulation. In the heart, TTCC is found in the sinoatrial nodal (SAN) and conduction cells. Cav3.1 encodes one of the three types of TTCCs. To date, there is no report regarding the regulation of Cav3.1 by β-adrenergic agonists, which is the topic of this study. Ventricular myocytes (VMs) from Cav3.1 double transgenic (TG) mice and SAN cells from wild type, Cav3.1 knockout, or Cav3.2 knockout mice were used to study β-adrenergic regulation of overexpressed or native Cav3.1-mediated T-type Ca^2+^ current (I_Ca-T(3.1)_). I_Ca-T(3.1)_ was not found in control VMs but was robust in all examined TG-VMs. A β-adrenergic agonist (isoproterenol, ISO) and a cyclic AMP analog (dibutyryl-cAMP) significantly increased I_Ca-T(3.1)_ as well as I_Ca-L_ in TG-VMs at both physiological and room temperatures. The ISO effect on I_Ca-L_ and I_Ca-T_ in TG myocytes was blocked by H89, a PKA inhibitor. I_Ca-T_ was detected in control wildtype SAN cells but not in Cav3.1 knockout SAN cells, indicating the identity of I_Ca-T_ in normal SAN cells is mediated by Cav3.1. Real-time PCR confirmed the presence of Cav3.1 mRNA but not mRNAs of Cav3.2 and Cav3.3 in the SAN. I_Ca-T_ in SAN cells from wild type or Cav3.2 knockout mice was significantly increased by ISO, suggesting native Cav3.1 channels can be upregulated by the β-adrenergic (β-AR) system. In conclusion, β-adrenergic stimulation increases I_Ca-T(3.1)_ in cardiomyocytes_,_ which is mediated by the cAMP/PKA pathway. The upregulation of I_Ca-T(3.1)_ by the β-adrenergic system could play important roles in cellular functions involving Cav3.1.

## Introduction

T-type Ca^2+^ channels (TTCCs or Cav3) belong to one of the families of voltage-dependent Ca^2+^ channels. These channels are activated and inactivated at low membrane potentials (the threshold is about −60 mV) with rapid time-dependent decay (**t**ransient) and **t**iny single channel currents and thus termed **T**-type. They are encoded by three genes, Cav3.1 (α1G), Cav3.2 (α1H) and Cav3.3 (α1I) [1], [2], [3], [4], [5]. The identification of the genes encoding TTCCs [2], [3], [5] allows the examination of the properties, distribution and function of each subtype of TTCCs and offers the potential to design isoform-specific TTCC antagonists to treat related channelopathies.

TTCCs are present in a wide variety of tissues including the heart, brain, skeletal muscle, testis and spermatids, indicating multiple functions of these channels such as cardiac rhythm generation, neuronal excitability, hormone secretion, neurotransmitter release, vascular tone regulation, muscle contraction, gene expression, cell metabolism, differentiation, and proliferation [2], [3], [5], [6]. Therefore, abnormal expression and function of TTCCs are associated with many diseases including cardiac hypertrophy and arrhythmia, hypertension, epilepsy, autism, and cancer [6].

TTCCs are expressed in the whole heart during the embryonic stage but their expression in the ventricle decreases rapidly after birth [7]. Cav3.1 and Cav3.2 expression is retained in the sinoatrial node (SAN), atrioventricular node (AVN) and Purkinje fibers of the adult heart, indicating a role in cardiac automaticity and conduction [7]. Mice deficient of Cav3.2 showed normal sinoatrial rhythm [8], but mice lacking Cav3.1 had prolonged SAN recovery time, slowed pacemaker activity of SAN cells and heart rate, and delayed atrioventricular conduction. These results indicate Cav3.1, rather than Cav3.2, is the major TTCC participant in cardiac rhythm generation in the mouse heart [9].

Since β-adrenergic system is critical for heart rate regulation and Cav3.1 is involved in cardiac rhythm generation, it is important to examine the regulation of the TTCC by the β-adrenergic/PKA system. The regulation of TTCCs by cAMP-dependent protein kinase A (PKA) has been controversial probably due to the differences in experimental conditions, cell types and the existence of specific isoforms [10]. In general it is believed that PKA has little effects on TTCCs [11], [12], [13]. Phosphorylation of Cav3.2 by PKA has been shown to permit the inhibitory effect of Gβγ dimmers [14]. In contrast, T-type Ca^2+^ current (I_Ca-T_, probably through Cav3.2 because it was sensitive to low concentration of Ni^2+^) in frog atrial myocytes was reported to be increased by isoproterenol via a cAMP/PKA independent mechanism [15]. The same group showed that cAMP/PKA downstream to β-adrenergic receptor might phosphorylate a protein to enhance high-voltage prepulse-induced facilitation of TTCCs [16]. In addition, Lenglet et al. also reported that Cav3.2 TTCC activity recorded in rat glomerulosa cells was augmented by PKA after the stimulation of 5HT7 receptors [17]. To date, there is no report of the regulation of Cav3.1 by the β-adrenergic receptor/cAMP/PKA cascade in cardiac or other native mammalian cells.

In this study, we sought to determine whether Cav3.1 is regulated by β-adrenergic receptor/PKA signaling pathway using ventricular myocytes from Cav3.1 transgenic mice and sinoatrial node cells from wildtype or Cav3.2 knockout mice. We have found that the activity of both overexpressed and native Cav3.1 channel is enhanced by a β-adrenergic agonist, isoproterenol. This effect was mediated by the adenylyl cyclase/cAMP/PKA system because cAMP recapitulated the effect of isoproterenol while a PKA inhibitor (H89) abolished the effect of ISO on I_Ca-T(3.1)_. The upregulation of Cav3.1 by PKA may contribute to the regulation of the heart rate by the β-adrenergic system.

## Materials and Methods

### Ethical Approval

This study conforms to the *Guide for the Care and Use of Laboratory Animals* published by the US National Institutes of Health (NIH Publication No. 85-23, revised 1996) and was approved by the Institutional Animal Use and Care Committee at Temple University.

### Animal Models

A mouse model with cardiac-specific (α-MHC controlled) and conditional (tet-off, controlled by doxycycline, DOX) overexpression of mouse Cav3.1 (Genebank: NM_009783) was established with the bitransgenic system developed by Sanbe A et al [18]. Cav3.1 transgenic (TG) mice were used for isolating ventricular myocytes (VMs). Cav3.1 knockout and Cav3.2 knockout mouse colonies were obtained from the Molkentin’s group [19] for isolating the SAN cells.

### Ventricular Myocyte and Sinoatrial Node Cell Isolation

Mouse ventricular myocytes (VMs) were isolated using a constant-pressure Langendorff apparatus as described [20]. Animals were anesthetized with sodium pentobarbital (120 mg/kg body weight). The heart was excised and digested via retrograde perfusion of the heart with normal Tyrode solution containing type II collagenase (290 U/mL) and (in mM): CaCl_2_ 0.02, glucose 10, HEPES 5, KCl 5.4, MgCl_2_ 1.2, NaCl 150, sodium pyruvate 2 (pH 7.4 with NaOH). After 8–10 min, the ventricles were minced and isolated ventricular myocytes were dissociated by gentle aspiration of minced tissue. Isolated VMs then were filtered with a 200 µm-diameter mesh. VMs were maintained in normal Tyrode solution containing 0.5% bovine serum albumin and the extracellular Ca^2+^ was titrated up to 1 mM. Myocytes were used within 8 hours after isolation.

SAN cells were isolated as described previously [21], [22]. Animals were anesthetized with sodium pentobarbital (120 mg/kg BW, intraperitoneal injection) and heparinized intravenously. SAN cells were isolated with a “chunk” technique as follow: After the heart was excised, it was placed into Tyrode’s solution (35°C) containing (in mM) 140 NaCl, 5.0 HEPES, 5.5 Glucose, 5.4 KCl, 1.8 CaCl_2_, 1.0 MgCl_2_ (pH = 7.4). The SAN region was the one with spontaneous activity and where the contraction of the heart initiates. The SAN tissue was dissected out with a dissecting scope according to the landmarks of the heart (delimited by the orifice of superior vena cava, crista terminalis and atrial septum) as described [21], [22]. The SAN tissue was cut into smaller pieces, which were transferred and rinsed in a “low Ca^2+^” digestion containing (mM) 140 NaCl, 5.0 Hepes, 5.5 Glucose, 5.4 KCl, 0.2 CaCl_2_, 0.5 MgCl_2_, 1.2 KH_2_PO_4_, 50 Taurine, and 1 mg/mL BSA (pH = 6.9). Then SAN tissue pieces were digested in 5 mL of “low Ca^2+^” solution containing collagenase type I (Worthington, 225 U/mL), elastase (Worthington, 1.8 U/mL), and protease type XIV (0.8 U/mL, Sigma) for 30 min at 37°C. After digestion, the tissue was washed with 10 mL of Kraft-Bruhe medium containing (mM) 100 potassium glutamate, 5.0 Hepes, 20 Glucose, 25 KCl, 10 potassium aspartate, 2.0 MgSO_4_, 10 KH_2_PO_4_, 20 taurine, 5 creatine, 0.5 EGTA, and 1 mg/mL BSA (pH = 7.2) for 3 times and then the cells were dissociated with a transfer pipette by pipetting up and down the tissue chunks. Dissociated cells were put at room temperature for 5 min. The cells were stored at 4°C and studied within 5 hours.

### Electrophysiology

**Figure 1:**
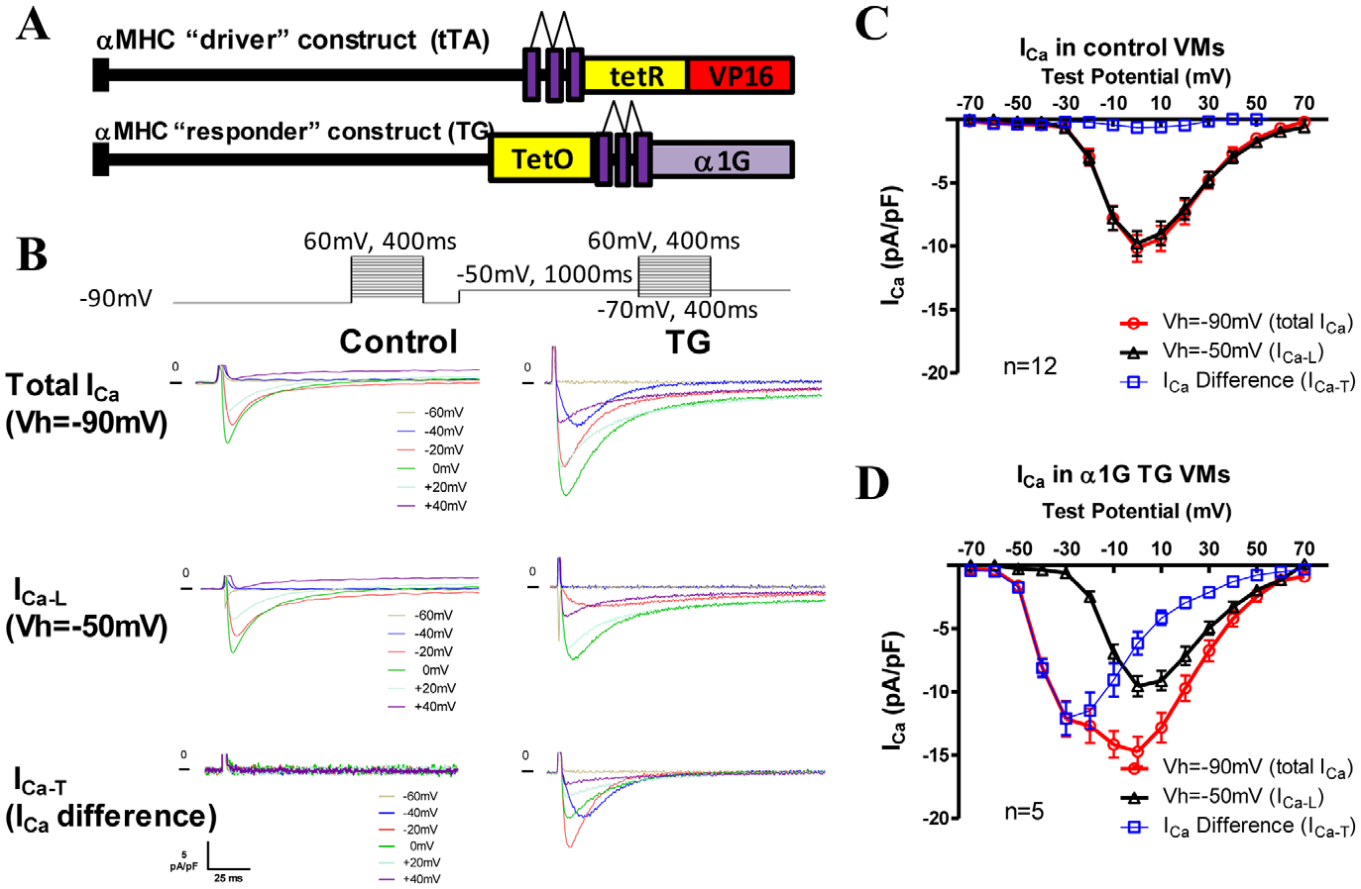
I_Ca-T(3.1)_ was expressed only in Cav3.1 TG ventricular myocytes and sensitive to mibefradil but not to dihydropyridines. **A**, A schematic of the bitransgenic inducible expression system for cardiac-specific Cav3.1 overexpression. tTA is the tetracycline-controlled transactivator system. tetR, tet-repressor cDNA fused to VP16 (activator domain); tet-O, tet-operon. **B**, Raw currents of total I_Ca_, I_Ca-L_ and I_Ca-T_ in a control and a TG VMs. No I_Ca-T_ was present in this control cell but robust I_Ca-T_ found in the TG VM. **C & D**, Averaged I–V curves of total I_Ca_, I_Ca-L_ and I_Ca-T_ in control (n = 12) and TG (n = 13) ventricular myocytes. No I_Ca-T_ was detected in control VMs but great I_Ca-T_ found in TG VMs.

Ca^2+^ currents (I_Ca_) were measured using whole-cell voltage-clamp techniques with an Axopatch 2B voltage-clamp amplifier and pClamp8 software and 1–3 MΩ pipettes. Ca^2+^ currents were measured under discontinuous voltage-clamp mode. The real clamping voltage and Ca^2+^ currents were measured simultaneously. To achieve good voltage control, the gain was set between 8 to 50. To ensure the quality of our data, only data with the loss of voltage control <10 mV were included for our report. VMs were placed in a heated chamber (35±2°C or room temperature) on the stage of an inverted microscope (Nikon Diaphot, Japan) and initially perfused with normal Tyrode solution. The pipette contained a Na^+^-free and K^+^-free solution consisting of (in mM): Cs-aspartic acid 130, EGTA 10, MgCl_2_ 1, NMDG 10, HEPES 10, TEA-Cl 20, Tris-ATP 5, Tris-GTP 0.25, pH 7.2. Once a gigaohm seal was obtained, the patched membrane was ruptured to allow 10 minutes of dialysis of the myocyte. The perfusate was switched to a Na^+^-free, K^+^-free solution containing (in mM): CaCl_2_ 2, 4-aminopyridine (4-AP) 2, CsCl 5.4, Glucose 10, HEPES 5, MgCl_2_ 1.2, NMDG 150, pH 7.4. Myocyte capacitance was obtained with the membrane test function in Clampex 8.0, which hyperpolarizes the cell for −5 mV from the holding potential to measure cell capacitance. The total calcium current (I_Ca_)-voltage relationship was determined by measuring I_Ca_ from a holding potential of −90 mV using square wave pulses in a 10 mV- increment. I_Ca-L_ was measured at the same test voltages from a holding potential of −50 mV. To minimize run-down of Ca^2+^ currents, these two Ca^2+^ currents measured at the same test potential were separated by a 1000 ms holding potential of −50 mV (**Figure 1B**) and 20 s was set between sweeps. I_Ca-T_ at each test membrane potential was determined by subtracting raw I_Ca-L_ from raw total I_Ca_ at this test potential, which is a traditional way for measuring I_Ca-T_ [1]. Mibefradil (1 µM) or BayK 8644 (1 µM) was applied via perfusate to the cell while the Ca^2+^ currents measured at the test potential −40 mV from the Vh of −90 mV and at 10 mV from the Vh of −50 mV to 10 mV were continuously monitored. BayK 8644, a DHP agonist, was selected because it could readily and affirmatively determine if BayK 8644 had any stimulatory effect on I_Ca-T_ even in the presence of a rundown of I_Ca-T_.

To determine ISO effect on the current-voltage (I–V) relationships of I_Ca-L_ and I_Ca-T_, I–V relationships of total I_Ca_ and I_Ca-L_ were recorded at baseline. Subsequently, 1 µM isoproterenol (ISO, Sigma) were applied through perfusate while the currents elicited from the Vh = −90 mV to −40 mV (mostly I_Ca-T_) and from the V_h_ = −50 mV to 10 mV (mostly I_Ca-L_) were monitored until the maximum effect of ISO was observed. Then, the I–V relationships of total I_Ca_, I_Ca-L_ and I_Ca-T_ were determined again as described above. To determine if direct activation of PKA was able to regulate I_Ca-T_, a nonhydrolyzable cAMP analog (dibutyryl-cAMP, Sigma, 10 µM) was dialyzed into the cell for 10 minutes and the I_Ca-L_ and I_Ca-T_ were determined afterwards [23]. To determine if ISO effects on I_Ca-L_ and I_Ca-T_ is mediated by PKA, a PKA inhibitor (H89, Sigma, 5 µM), was included in the pipette filling solution and patched VMs were dialyzed for 10 minutes. Thereafter, the I–V relationships of total I_Ca_, I_Ca-L_ and I_Ca-T_ were measured before and after the application of ISO.

To investigate the effects of ISO and db-cAMP on voltage-dependent inactivation of I_Ca-L_ and I_Ca-T(3.1)_, double-pulse protocols were applied with or without these drugs. For I_Ca-T(3.1)_, the first pulse was the prepulse to different test voltages (−110 mV to −10 mV in a10 mV-increment) from the holding potential −90 mV. The second pulse was separated by a 5 ms repolarizing period to −90 mV from the prepulses and depolarized to −40 mV, a voltage almost only I_Ca-T_ and minimal I_Ca-L_ contamination could be recorded. For I_Ca-L_, the prepulses were evoked from the holding potential of −50 mV to different membrane potentials and the second pulse was evoked from a short return to −50 mV for 5 ms to 0 mV (for 400 ms).

Since in small SAN cells, rundown of I_Ca-L_ and I_Ca-T_ was faster (often I_Ca-T_ was decreased by >30% within 10 minutes), it became a significant issue to study ISO effects if a long recording time was needed. Full I–V curves before and after ISO could not obtained. Therefore, to study ISO effects on I_Ca-T_ in SAN cells, we only studied ISO effect at a single voltage (from the holding potential of −90 mV to −40 mV) in the presence of nifedipine (an I_Ca-L_ blocker, 10 µM). The use of nifedipine ensured only I_Ca-T_ was recorded at this voltage even ISO could shift the activation of I_Ca-L_ to more negative voltage and thus avoided the use of double pulses to minimize recording time to minimize I_Ca-T_ rundown.

The conductance of L- and T-type Ca^2+^ channels (G_Ca_) was calculated by dividing the current by the driving force (V_t_-E_Ca_’, where E_Ca_’ stands for the apparent reversal potential of Ca^2+^). The activation-voltage (G-V) relationships were plotted using normalized L- or T-type Ca^2+^ channel conductance (G_Ca_/G_Ca,max_) as the y-axis and test potentials as the x-axis. The G-V curve is fitted with a Boltzmann function G/G_max_ = 1/[1+exp[−(V_t_−V_0.5, d∞_)/*k*]], where V_t_ is the test voltage and V_0.5, d∞_is the voltage at which half of the G_max_ can be elicited, *k* is the slope factor. The voltage-dependent inactivation curve (f_∞_) was fitted with a Boltzmann function G/G_max_ = 1/[1+exp[−(V_t_−V_0.5, f∞_)/*k*]], where V_t_ is the test voltage and V_0.5, f∞_is the voltage at which half of the channels were inactivated, *k* is the slope factor.

### Real-time PCR

SAN tissue was pooled from 4–8 mice for wild type (n = 24) and transgenic (n = 19) animals. Total mRNA was extracted from snap-frozen SAN tissue, ventricular tissue, or brain (as a positive control for T-type Ca^2+^ channel expression) using Trizol reagent and quantitated by a UV spectrometer. Real-time PCR was done with the SYBR Green Real Time PCR kit (Applied Biosystems, Carlsbad, CA) according to the instruction of the kit and an Eppendorff Mastercycler RT-PCR machine. GAPDH was used as the internal control. The ΔΔCt-method was used to determine the abundance of Cav3 mRNAs relative to GAPDH. The primers were (5′ to 3′): **Cav3.1**: forward: TGTGGAAATGGTGGTGAAGA and reverse: ACTGCGGAGAAGCTGACATT; **Cav3.2**: forward: GCTGTTTGGGAGGCTAGAAT and reverse: CGAAGGTGACGAAGTAGACG; **Cav3.3**: forward: TGGGCATTTTTGGCAAGAA and reverse: CAGTGCGGATGGCTGACA; **GAPDH**: forward: TGCACCACCAACTGCTTAG and reverse: GATGCAGGGATGATGTTC.

### Data Analysis

Obtained data were analyzed offline with Clampfit 8 (Molecular Device, CA) as described previously [23], managed with Microsoft Excel and presented with GraphPad Prizm 5.0 (La Jolla, CA, USA). In short, I_Ca-T_ was obtained by subtracting the raw I_Ca-L_ from the raw total I_Ca_ as described previously [24]. The current-voltage (I–V) relationships were constructed by plotting the peak amplitudes of total I_Ca_, I_Ca-L_ and derived I_Ca-T_ against the test voltages.

### Statistics

Data in the text are reported as mean±SEM. When appropriate, paired and unpaired T-test, ANOVA or ANOVA for repeated measures were used to detect significance with SAS 9.0 (SAS Institute Inc.). P values of ≤0.05 were considered significant. In this paper, *n* is the number of cells examined from at least 3 animals.

## Results

### Cav3.1 Mediated T-type Ca^2+^ Current (I_Ca-T(3.1)_) Was Observed Only in VMs from Cav3.1 Double Transgenic Mice

To examine whether Cav3.1 was expressed in our transgenic system, I_Ca-T_ was measured in VMs from transgenic (TG) and control hearts. Typical examples of total Ca^2+^ current (I_Ca_), L-type Ca^2+^ current (I_Ca-L_) and T-type Ca^2+^ current (I_Ca-T_) in control and TG VMs are shown in **Figure 1B**. The current-voltage relationships **(**I–V curves) of total I_Ca_, I_Ca-L_ and I_Ca-T_ in both control (n = 12) and TG myocytes (n = 5) are shown in **Figure 1C** and **D**. There was no detectable I_Ca-T_ observed in all 12 tested control cells (**Figure 1B and C**) but a great density of I_Ca-T_ (maximum I_Ca-T_ amplitude: −12.1±1.3pA/pF) was found in Cav3.1 TG VMs (**Figure 1B and D**). I_Ca-T_ had a threshold of ∼−60 mV and peaked at ∼−40 mV. The time-dependent decay rates (kinetics) of the I_Ca-T_ were significantly faster than those of the I_Ca-L_ recorded in the same cell (**Figure 1B**), as reported previously [1].

**Figure 2:**
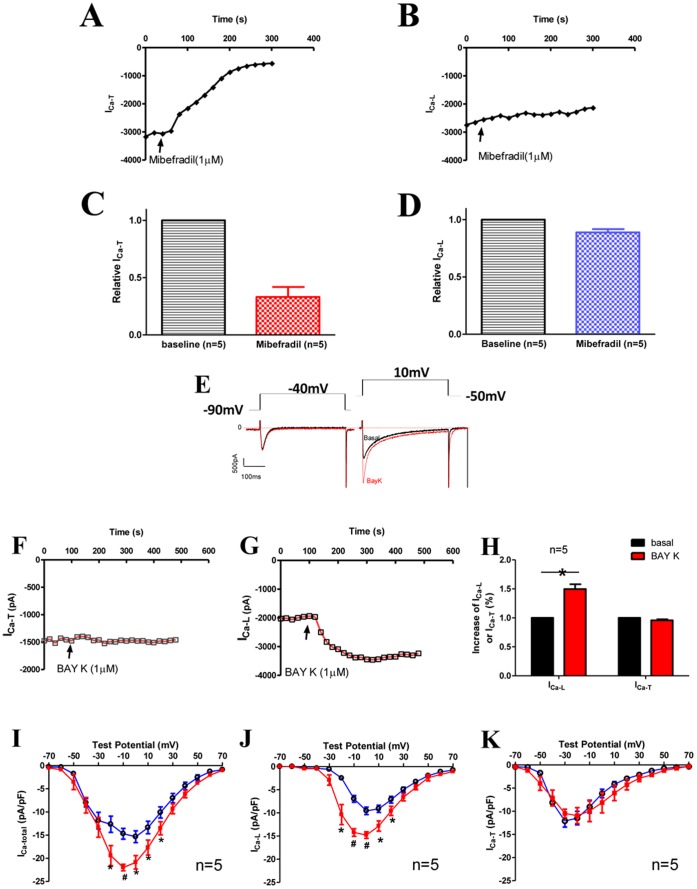
I_Ca-T(3.1)_ in Cav3.1 TG ventricular myocytes was sensitive to mibefradil but not to dihydropyridines. **A** & **B**, Time courses of amplitude changes of presumable I_Ca-L_ and I_Ca-T_ in response to mibefradil. **C & D**, Relative changes of ICa-T (**C**) and ICa-L (**D**) in response to mibefradil. **E**, Raw currents recorded at −40 mV from Vh = −90 mV and at 10 mV from Vh = −50 mV before and after the application of BayK 8644. **F & G**, Time courses of amplitude changes of presumable I_Ca-L_ and I_Ca-T_ in response to BayK. **H**, Normalized increases in presumable I_Ca-L_ and I_Ca-T_ by BayK. **I**, **J and K**, I–V curves of total I_Ca_, I_Ca-L_ and I_Ca-T_ before and after BayK.

To confirm the identity of putative I_Ca-T_, mibefradil (1 µM) was used to determine its sensitivity to this TTCC antagonist. Mibefradil suppressed I_Ca-T_ at −40 mV by 66.8±8.7% (**Figure 2A and C**) but had minimal effect on I_Ca-L_ at 10 mV (decreased by 11.1±2.8%; **Figure 2B and D**). To further confirm that I_Ca-T_ could be effectively separated by holding the cell at different membrane potentials with minimal contamination of I_Ca-L_, BayK 8644, a dihydropyridine agonist specific for I_Ca-L_, was used to test dihydropyridine effect on the two components of I_Ca_. As suggested in **Figure 1C** and **D**, I_Ca_ recorded at −40 mV from V_h_ of −90 mV was almost completely consisting of I_Ca-T_ while I_Ca_ recorded at 10 mV from V_h_ of −50 mV should be only I_Ca-L_. As predicted, I_Ca_ at −40 mV from the V_h_ of −90 mV was not changed by BayK (**Figure 2E, F and H**) while I_Ca_ at 10 mV from the V_h_ of −50 mV was significantly increased 49.7±0.8% by BayK (**Figure 2E, G and H**). When the I–V curves of presumable I_Ca-L_ and I_Ca-T_, separated by the strategy of holding the cell at different membrane potentials, were examined, BayK evidently increased the presumable I_Ca-L_ but not the presumable I_Ca-T_ (**Figure 2I, J and K**). These results suggest that the separation of I_Ca-L_ and I_Ca-T_ by holding the cell at different membrane potentials is effective, and that I_Ca-T_ is not sensitive to dihydropyridines but sensitive to mibefradil.

### ISO Significantly Increased I_Ca-T(3.1)_ in TG VMs

**Figure 3:**
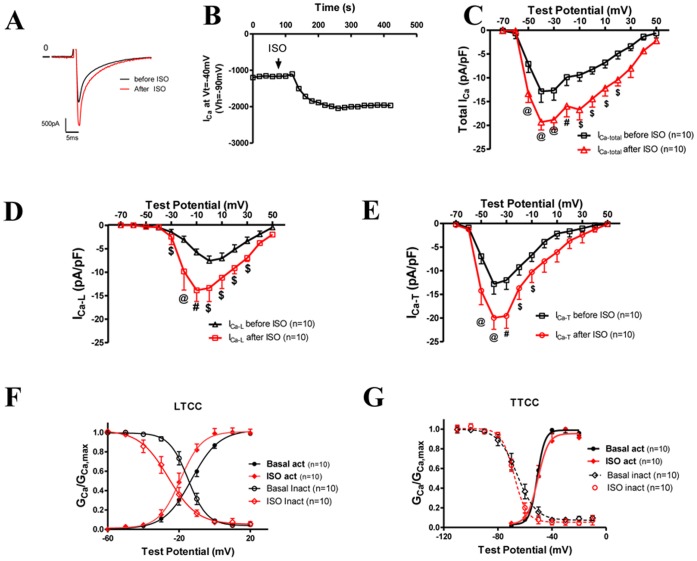
ISO significantly increased I_Ca-T(3.1)_ and I_Ca-L_. **A**, Raw current recordings at −40 mV from the V_h_ of −90 mV before and after ISO. **B**, Time course of the change of the amplitude of I_Ca_ measured at the test potential of −40 mV from the V_h_ = −90 mV, which was almost completely consisting of I_Ca-T(3.1)_. **C-E**, Averaged I–V curves of total I_Ca_ (**C**), I_Ca-L_ (**D**), and I_Ca-T_ (**E**) before and after ISO application. ISO increased total I_Ca_, I_Ca-T_ and I_Ca-L_ at most membrane potentials. ISO shifted the voltage at which I_Ca-L_ peaked from 0 mV to −10 mV but did not change the voltage (−40 mV) at which I_Ca-T(3.1)_ peaked. **F** and **G**: the voltage-dependent activation and inactivation curves of I_Ca-L_ and I_Ca-T_ before and after the application of ISO. @: *p<0.001;* #: *p<0.01; $: p<0.05;* TG vs. control at the same test potential. Statistics were done with two-way ANOVA and post-hoc t-test.

To study whether I_Ca-T(3.1)_ can be regulated by the β-adrenergic system in cardiac myocytes, isoproterenol, a β-adrenergic agonist, was applied to TG VMs. Since I_Ca_ at −40 mV from the V_h_ of −90 mV was almost completely consisting of the T-type Ca^2+^ current, ISO effect on I_Ca-T(3.1)_ was monitored by recording I_Ca_ at −40 mV. **Figure 3A** showed I_Ca_ at −40 mV before and after ISO and Figure **3B** showed the time course of the ISO effect on a myocyte, suggesting a significant upregulation of I_Ca-T(3.1)_. I–V curves of total I_Ca_ (**Figure 3C**), I_Ca-L_ (**Figure 3D**), and I_Ca-T_ (**Figure 3E**) before and after ISO showed that β-adrenergic stimulation (ISO) significantly increased both I_Ca-L_ and I_Ca-T_ at most test voltages. The maximal I_Ca-L_ was increased by 81.6% (from −7.6±1.2pA/pF to −13.8±1.3pA/pF) and the maximal I_Ca-T(3.1)_ was increased by 55.5% (from −12.8±1.9pA/pF to −19.9±2.4pA/pF). It is well known that β-adrenergic stimulation shifts the activation of I_Ca-L_ to more negative voltages and accelerates the decay of I_Ca-L_ via a Ca^2+^-dependent inactivation mechanism [24]. In this study, we found that ISO shifted the peak I_Ca_ voltage for I_Ca-L_ but not for I_Ca-T(3.1)_ (**Figure 3D and E**). It seems that there is no voltage dependent effect of ISO because ISO did not change the voltage-dependence of I_Ca-T(3.1)_ activation and inactivation although it shifted the activation and inactivation of I_Ca-L_ to more negative voltages (**Figure 3F and 3G** **)**.

### Nonhydrolyzable Dibutyryl-cAMP (db-cAMP) Significantly Increased I_Ca-T(3.1)_ at Both Physiological and Room Temperatures

**Figure 4:**
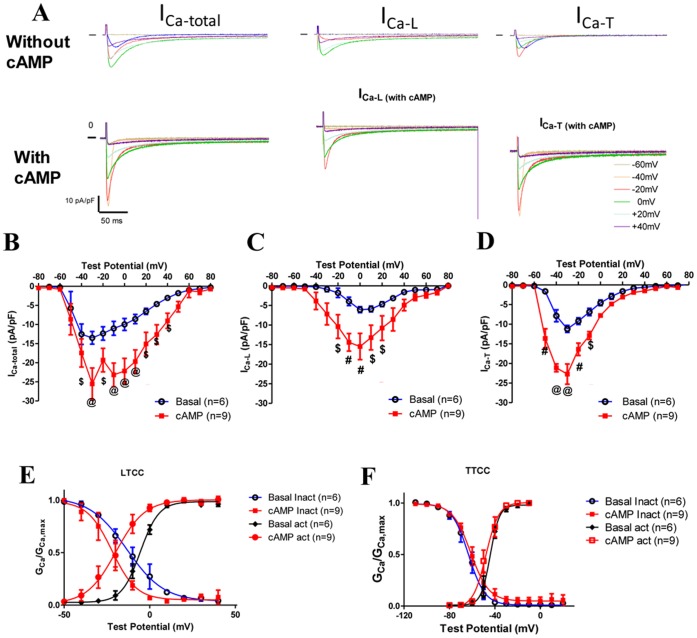
A nonhydrolyzable cAMP analog, db-cAMP, significantly increases I_Ca-T_ and I_Ca-L_. **A**, Example currents of total I_Ca_, I_Ca-L_ and I_Ca-T_ without (top) or with db-cAMP (lower) in the pipette recorded in TG VMs. Currents were normalized to cell capacitance for comparison and the scale bars are the same for both without or with db-cAMP recordings. **B–D**, Averaged I–V curves of total I_Ca-total_ (**B**), I_Ca-L_ (**C**) and I_Ca-T_ (**D**) with or without db-cAMP in the pipette. There are significantly increases in both I_Ca-L_ and I_Ca-T(3.1)_. Db-cAMP shifted the peak voltage of I_Ca-L_ to more negative voltages but did not change that of I_Ca-T(3.1)_ in TG VMs. **E** and **F**: the voltage-dependent activation and inactivation curves of I_Ca-L_ and I_Ca-T_ with or without db-cAMP. @: *p<0.001;* #: *p<0.01; $: p<0.05;* TG vs. control at the same test potential. Statistics were done with two-way ANOVA and post-hoc t-test.

The stimulatory effect of β-adrenergic agonists on calcium channels in cardiac myocytes can be mediated by mulitple mechanisms [24]: 1, PKA activation through the β-adrenergic receptor/Gαs/adenyl cyclase/cAMP/PKA pathway; 2, direct modulation by Gαs or Gβγ after the binding of adrenergic agonists to the adrenergic receptor and subsequent dissociation of Gαs from Gβγ dimer; 3. The newly found cAMP sensor, EPAC (exchange protein directly activated by cAMP), regulates I_Ca-T_. We tested if direct activation of PKA by a cAMP analog (db-cAMP) was able to stimulate I_Ca-T(3.1)_. Db-cAMP significantly increased I_Ca-L_ by 154.9% (with db-cAMP −15.5±3.3pA/pF versus without db-cAMP −6.1±0.8pA/pF, **Figure 4B**) and increased I_Ca-T(3.1)_ by 102.8% (with db-cAMP 22.7±2.6pA/pF vs. without db-cAMP −11.2±0.9pA/pF, **Figure 4C**). The voltage-dependence of I_Ca-T_ activation and inactivation was not altered by db-cAMP (**Figure 4F**) although db-cAMP shifted the activation curve and inactivation curve to the left (**Figure 4E**). These findings clearly show that the ISO effects of I_Ca-T(3.1)_ are recapitulated by cAMP, indicating that the upregulation of I_Ca-T(3.1)_ by ISO is mediated by the cAMP dependent PKA signaling pathway.

### The Effect of ISO on I_Ca-T (3.1)_ can be Blocked by a PKA Inhibitor, H-89

**Figure 5:**
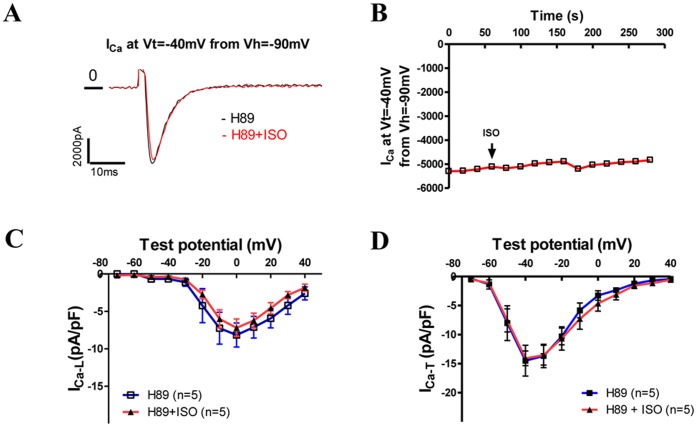
The effect of ISO on I_Ca-T_ and I_Ca-L_ can be blocked by a PKA Inhibitor, H89. **A**, Raw currents evoked from the holding potential of −90 mV to −40 mV before and 4 minutes after the application of ISO in a TG myocyte dialyzed with pipette H89 (10 µM). **B**, The time course of the change of the amplitude of I_Ca-T(3.1)_ (recorded at V_t_ = −40 mV from the V_h_ = −90 mV). H89 blocked the effect of ISO on I_Ca-T(3.1)_. **C &** **D**, Averaged I–V curves of I_Ca-L_ and I_Ca-T_ in TG VMs dialyzed with H89 before and after the application of ISO.

Albeit our results with db-cAMP imply that the stimulatory effect of ISO might be mediated by PKA, recently another cAMP sensor, an exchange protein directly activated by cAMP (EPAC), has been found in cells from various tissues including the heart [25]. To rule out the involvement of EPAC and confirm the role of PKA in the stimulatory effects of ISO, a PKA-specific inhibitor, H89, was included in the pipette filling solution. In the presence of H89, ISO did not increase the current recording at the Vt = −40 mV from Vh = −90 mV (mainly I_Ca-T(3.1)_) before and after the application of ISO (raw currents in **Figure 5A** and time course in **Figure 5B**). I–V curves of I_Ca-L_ and I_Ca-T_ were not changed by ISO with H89 in the pipette filling solution (**Figure 5C & D**). These results support the idea that PKA activated by β-adrenergic agonists causes the upregulation of I_Ca-T(3.1)_.

### I_Ca-T(3.1)_ is the T-type Ca^2+^ Current in Mouse SAN Cells and is Upregulated by Isoproterenol

**Figure 6:**
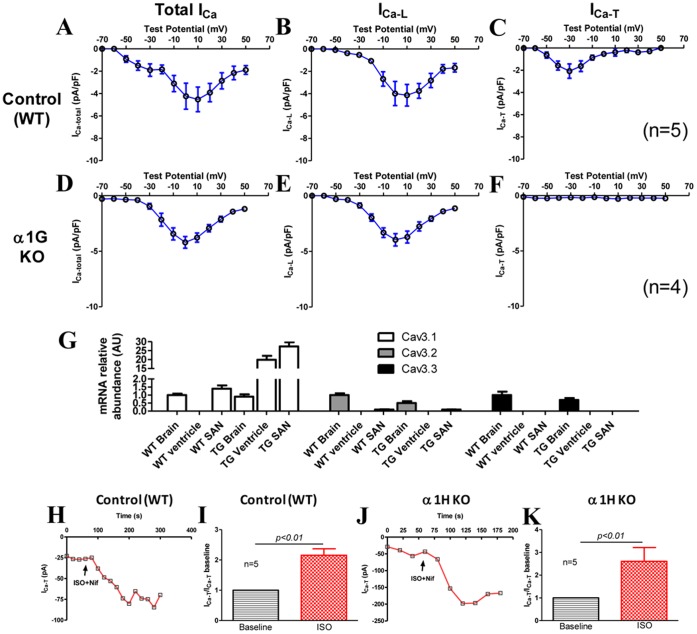
I_Ca-T_ in mouse SAN cells is mediated by Cav3.1 and can be increased by ISO. I–V curves of total I_Ca_, I_Ca-L_ and I_Ca-T_ in wild-type control (**A, B and C**) and in Cav3.1 knockout (**D, E and F**) SAN cells were shown in **A-F**, showing I_Ca-T_ is present in WT cells but not Cav3.1 KO cells. **G**, mRNA abundance of Cav3.1, Cav3.2, Cav3.3 in wildtype brain, wildtype ventricle, wildtype SAN, Cav3.1 TG brain, Cav3.1 TG ventricle, and Cav3.1 TG SAN, measured by real-time PCR. **H and J**, time courses of the change of I_Ca-T_ (recorded at −40 mV in the presence of nifedipine) amplitudes by ISO+nifedipine in control wildtype (**H**) and Cav3.2 KO (**J**) SAN cells. **I and K**, normalized increases of I_Ca-T_ (recorded at −40 mV in the presence of nifedipine) by ISO+nifedipine in control wildtype (**I**) and Cav3.2 KO (**K**) SAN cells.

At last, we examined if native I_Ca-T_ in SAN cells is regulated by the β-adrenergic system. Total I_Ca_, I_Ca-T_ and I_Ca-L_ were recorded from wild type and Cav3.1 KO SAN cells. In wild type SAN cells, I_Ca-T_ was clearly recorded with a maximum current of −2.08±0.64pA/pF (**Figure 6** **A–C**). In contrast, in the SAN cells from Cav3.1 knockout mice, T-type Ca^2+^ current is completely absent, indicating that Cav3.1 is the major mediator of I_Ca-T_ in mouse SAN cells (**Figure 6** **D–F**) as previous study suggests [9]. Real-time PCR further confirmed that the major TTCC in the SAN is Cav3.1. Cav3.2 mRNA abundance was very low and Cav3.3 mRNA was not detectable in SAN tissue although all three types of TTCCs were detected in the brain (**Figure 6** **G**). I_Ca (_presumably I_Ca-T_) measured at −40 mV in wild-type SAN cells bathed in 10 µM nifedipine (to block I_Ca-L_) was increased by 115.7±21.1% with 1 µM ISO (**Figure 6** **H and I**). To further make sure that I_Ca-T_ in SAN cells was Cav3.1-mediated and could be stimulated by 1 µM ISO, I_Ca-T_ was recorded in Cav3.2 knockout SAN cells and ISO did increase I_Ca-T_ in these cells by 161.0±20.5% (**Figure 6** **J and K**).

## Discussion

### What are the New Findings in this Study?

First, we found that β-adrenergic stimulation increased I_Ca-T(3.1)_ in cardiac myocytes expressing exogenous or endogenous Cav3.1 channels. To our knowledge, this is the first detailed study of β-adrenergic regulation of one specific subtype of TTCCs in cardiac myocytes thus far. Second, the cAMP/PKA pathway mediates the activity enhancing effect of β-adrenergic stimulation.

### The Regulation of TTCCs by the β-adrenergic/PKA Pathway

TTCCs are distributed in various cell types serving a wide range of functional roles [3], [26]. Abnormal expression of T-type Ca^2+^ channels is involved in pathological conditions including epilepsy [27], neurogenic pain [28], cancer [29], [30], and cardiac hypertrophy [31]. Therefore, the regulation of TTCCs has been studied since their discoveries [10]. However, early studies often show conflicting results possibly due to unknown molecular identities and TTCC heterogeneity in cells [10]. Interestingly, it has been shown that the same neurotransmitter, hormone, or protein kinase exerts different effects on subtypes of TTCCs or on the same TTCC subtype in different tissues [10]. In addition, in our study, we have observed a run-down phenomenon of I_Ca-T_ at baseline or during β-adrenergic stimulation in some myocytes, which could mask the stimulatory effects of β-adrenergic.

Specifically for β-adrenergic/PKA regulation of TTCCs, it remains controversial [32]. Most previous studies showed little effect of PKA on TTCCs [11], [12], [13]. Phosphorylation of Cav3.2 by PKA permits the inhibition of I_Ca-T_ by Gβγ dimmers [14]. On the other hand, T-type Ca^2+^ current has been reported be increase by ISO in frog atrial cells through a cAMP/PKA-independent mechanism [15] while in rat glomerulosa cells by 5-HT via a PKA-dependent mechanism [17]. Cav3.2 channel activity can be increased by db-cAMP in heterologous systems as well [32], [33]. To date, there has been no report about Cav3.1 regulation by β-adrenergic system, which is not present in heterologous systems. It has been shown that in the mouse heart, Cav3.1 is the primary TTCC found in SAN cells as our study also suggests [9]. Here, for the first time, we have shown that in cardiac myocytes only expressing Cav3.1 (TG myocytes or Cav3.2 knockout SAN cells), I_Ca-T(3.1)_ was significantly increased by ISO. Db-cAMP reproduces the effect of ISO on I_Ca-L(3.1)_ at both room and physiological temperatures while H89 (a PKA inhibitor) blocked ISO effect on I_Ca-L_ and I_Ca-T_ in TG myocytes, confirming that the stimulatory effect of ISO on I_Ca-L_ and I_Ca-T_ is mediated by PKA.

### Potential Mechanisms for PKA-dependent Upregulation of I_Ca-T(3.1)_

In the current study, we did not determine how I_Ca-T(3.1)_ is regulated by PKA activated by ISO. PKA might phosphorylate Cav3.1 directly as it does on L-type (Cav1) Ca^2+^ channels. Since it is generally believed that Cav3 channels do not have accessory subunits, the PKA site(s) could be on the Cav3.1α subunit. It is possible that PKA-dependent phosphorylation of Cav3.1 augments the open probability of the channel, as it does to the L-type Ca^2+^ channel. A second possibility is that PKA might phosphorylate another molecule to indirectly augment the open probability of the channel. Further studies will be needed to define the mechanism.

The effect of β-adrenergic stimulation on I_Ca-T_ could be reversed by dephosphorylation of Cav3.1α or a related protein if PKA phosphorylation is the mechanism for the upregulation of Cav3.1 activity. Multiple protein serine/threonine phosphatases including PP1, PP2A, and PP2B, are expressed in cardiac myocytes. Which phosphatase is involved in Cav3.1 by ISO warrants further investigation.

### Relevance of β-adrenergic Mediated Upregulation of I_Ca-T(3.1)_ in the Heart

In the heart, T-type calcium channels contribute to the heart rate generation [34], and it is possible that β-adrenergic/PKA regulation of Cav3.1 may participate in the positive chronotropic effects of β-adrenergic agonists. I_Ca-T_ is also reported to be reemerged and a participator in excitation-contraction coupling in stressed hearts; it is likely that β-adrenergic mediated upregulation of I_Ca-T_ might also contribute to cardiac contraction in stressed hearts although our previous studies have shown that overexpressed Cav3.1 is not effective to load the SR and trigger SR Ca^2+^ release [35]. Furthermore, if there is abnormally high I_Ca-T_ (e.g., overstimulation of the Cav3 channels by the sympathetic nervous system) in the cardiac pacemaking tissues and the conduction system, there could be tachycardia or atrial fibrillation or ectopic ventricular contraction. We have observed ectopic ventricular contraction in some Cav3.1 TG mice. In line with this, blocking Cav3 channels is able to reduce arrhythmic events and sudden cardiac death in a mouse heart failure model [36].

### Conclusion

In cardiac myocytes, Cav3.1 current is increased by β-adrenergic agonists. This effect is mediated by protein kinase A. The regulation of Cav3.1 by β-adrenergic/PKA signaling pathway could play a role in heart rate regulation, arrhythmias and regulating other cellular functions involving Cav3.1.
